# Chemistry in flow systems

**DOI:** 10.3762/bjoc.5.15

**Published:** 2009-04-29

**Authors:** Andreas Kirschning

**Affiliations:** 1 Institut für Organische Chemie and Zentrum für Biomolekulare Wirkstoffe (BMWZ), Leibniz Universität Hannover, Schneiderberg 1B, D-30167 Hannover, Germany

The environment in which synthesis is conducted has hardly changed over the last centuries: reactions are still typically performed batchwise in standardized glassware which has commonly been in use since the time of Justus von Liebig. In contrast, the use of flow-through processes is still rather restricted to the production site. Only recently have chemists in industry as well as in academia begun to focus on the development of flow devices for laboratory use and hence for industrial applications. An important field of research is the optimization and adaptation of known reactions and reaction sequences for use in flow systems. Advantageously, continuous-flow processes can be further improved by techniques that originate from high-throughput chemistry laboratories as they can be combined with the use of immobilized reagents or catalysts, or by using fixed bed reactors in parallel. These developments in flow techniques using mini and micro flow reactors have initiated changes that will pave the way for a technological step forward in chemical synthesis similar to that which took place in analytical chemistry and purification when chromatography started to conquer laboratories several decades ago, finally taking them by storm.

This **thematic series** on **chemistry in flow systems**, which includes original research papers and a review, mainly from the organic chemist’s perspective, will become part of this development, and we are happy to have assembled research by the leading research groups in this area from all over the world. I have to thank all contributing authors and colleagues who made this thematic series possible. It has been a great privilege and honour to assemble this magnificent crew of outstanding scientists, who worked to deadlines to put a lot of effort into the production of excellent and illustrative manuscripts. Particular thanks are directed to the Beilstein Journal of Organic Chemistry editorial and production team for their support and encouragement.

Hannover, April 2009

Andreas Kirschning

Guest Editor

